# Critical role for ERK1/2 in bone marrow and fetal liver–derived primary megakaryocyte differentiation, motility, and proplatelet formation

**DOI:** 10.1016/j.exphem.2009.07.006

**Published:** 2009-10

**Authors:** Alexandra Mazharian, Steve P. Watson, Sonia Séverin

**Affiliations:** Centre for Cardiovascular Sciences, Institute for Biomedical Research, College of Medical and Dental Sciences, University of Birmingham, Birmingham, UK

## Abstract

**Objective:**

Megakaryopoiesis and platelet formation is a multistep process through which hematopoietic progenitor cells develop into mature megakaryocytes (MKs) and form proplatelets. The present study investigates the regulation of different steps of megakaryopoiesis (i.e., differentiation, migration, and proplatelet formation) by extracellar signal-regulated kinase (ERK)1/2 and p38 mitogen-activated protein kinase (MAPK) in two models of primary murine MKs derived from bone marrow (BM) cells and fetal liver (FL) cells.

**Materials and Methods:**

A preparation of MKs was generated from BM obtained from femora and tibiae of C57BL6 mice. FL-derived MKs were obtained from the liver of mouse fetuses aged 13 to 15 days.

**Results:**

For both cell populations, activation of MEK-ERK1/2 pathway by thrombopoietin was found to have a critical role in MK differentiation, regulating polyploidy and surface expression of CD34, GPIIb, and GPIb. The MEK-ERK1/2 pathway plays a major role in migration of BM-derived MKs toward a stromal-cell−derived factor 1α (SDF1α) gradient, whereas unexpectedly, FL-derived cells fail to migrate in response to the chemokine due to negligible expression of its receptor, CXCR4. The MEK-ERK1/2 pathway also plays a critical role in the generation of proplatelets. In contrast, p38MAPK pathway was not involved in any of these processes.

**Conclusion:**

This report demonstrates a critical role of MEK-ERK1/2 pathway in MK differentiation, motility, and proplatelet formation. This study highlights several differences between BM- and FL-derived MKs, which are discussed.

Megakaryopoiesis is a tightly controlled multistep process of proliferation and differentiation involving commitment of hematopoietic multipotent progenitor cells to megakaryocyte (MK) precursors followed by maturation and (pro)platelet formation. During development, MKs undergo a series of transformations that can be identified by expression of surface proteins, including GPIIb (also known as the integrin subunit αIIb or CD41) and GPIb (CD42b), in association with nuclear maturation characterized by successive rounds of endomitosis and subsequent cytoplasmic maturation. The end result is large polyploid MKs, characterized by long, branching cytoplasmic extensions called proplatelets, which give rise to platelets [1−3].

Thrombopoietin (TPO) is a crucial regulator of megakaryocytic growth and differentiation in vitro and in vivo, exerting its effects through its receptor, c-Mpl [4−7]. c-Mpl signals via the Janus kinase/signal transducer and activator of transcription (JAK/STAT) [8] and Shc-Ras−mitogen-activated protein kinase (MAPK) pathways [9,10]. Several studies have reported a critical role for JAK2 and STAT5 in mediating MK development downstream of c-Mpl. Further, the V617F mutant of JAK2 is the causative mutation in approximately 50% of patients with the myeloproliferative disorder, essential thrombocythemia (ET), which is characterized by an increase in platelet count [11−13].

MAPKs are serine/threonine kinases that comprise extracellular signal-regulated kinases (ERKs), p38MAPKs and c-Jun amino-terminal kinases (JNKs) families [14], which are activated by dual phosphorylation of threonine and tyrosine residues. These three MAPK pathways are implicated in proliferation, survival, differentiation, and apoptosis of a wide variety of cells. The importance of the ERK1/2 pathway in MK differentiation was analyzed by expression of constitutively active or dominant-negative mutants of the upstream regulator of ERK1/2 kinases, MEK, and by use of pharmacological inhibitors of MEK (e.g., PD98059 and U0126) in immortalized megakaryocytic cell lines, including UT7-TPO [15], K562 [16−18], CMK [19], and in primary human MKs derived from cord or peripheral blood hematopoietic progenitor cells [20−23] and primary mouse bone marrow (BM)−derived MKs [24]. A general consensus is that the MEK-ERK1/2 pathway acts as a regulator of differentiation in MKs, principally promoting polyploidization in the later developmental stage [15−19,21,23,24]. Conflicting results on the role of MEK-ERK1/2 pathway on the differentiation of primary MKs have been published [20,22]. In addition, inhibition of ERK1/2 has been shown to increase [25], inhibit [26], or have no effect [27] on proplatelet formation in different MK models. These discrepancies may be due to the experimental conditions, the source of cells, or the concentration of the MEK inhibitors. In comparison, the role of the p38MAPK pathway in MK growth and differentiation has not been as extensively investigated and its various roles, if any, remain unclear [23,28,29].

This present study was undertaken to directly compare two primary mouse MK models derived from BM- and fetal liver (FL)-progenitor cells using established culture methods. The role of ERK1/2 and p38MAPK pathways in MK differentiation, migration, and proplatelet formation has been analyzed.

## Materials and methods

### Reagents, antibodies, and suppliers (detailed information can be found in Supplementary Methods)

#### MK purification and culture

**Supplementary Figure E1:**
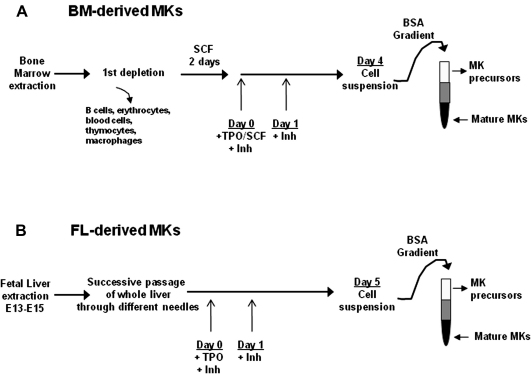
Bone marrow (BM)- and fetal liver (FL)-derived megakaryocyte (MK) purification and culture. (**A**) BM cells were obtained from femora and tibiae of C57BL6 mice by flushing and CD16/CD32^+^Gr1^+^B220^+^CD11b^+^ cells were depleted using immunomagnetic beads (sheep anti-rat IgG Dynabeads). The remaining population was cultured in 2.6% serum-supplemented Stempro medium with 2 mM l-glutamine, penicillin/streptomycin, and 20 ng/mL murine stem cell factor (SCF) at 37°C under 5% CO_2_ for 2 days. Cells were then cultured for an additional 4 days in the presence of 20 ng/mL murine SCF and 100 ng/mL murine thrombopoietin (TPO) [data from 30,32]. (**B**) FL cells were obtained from whole livers recovered from mouse fetuses between embryonic days 13 and 15. Single-cell suspensions were prepared by successive passage through 19-, 22-, and 25-gauge needles and cultured in Dulbecco's modified Eagle's medium supplemented with 10% fetal bovine serum, 2 mM l-glutamine, penicillin/streptomycin, and 100 ng/mL murine TPO for 5 days [data from 31]. After 4 to 5 days of culture in the presence of TPO, cell population was enriched on mature MKs after 1.5%/3% bovine serum albumin (BSA) gradient under gravity (1*g*) for 45 minutes at room temperature. The proportion of mature MKs in the enriched population was estimated to >60% (data not shown). To study the role of mitogen-activated protein kinase (MAPK) in differentiation, inhibitors (Inh) were added at the same time than TPO corresponding to the beginning of the differentiation process (day 0) and the inhibitor dose were repeated after 24 hours (day 1).

Mature MKs from BM- and FL- cells were defined as the population of cells generated using the methodology of the Frampton and Shivdasani laboratories, respectively [30,31]. In brief, BM cells were obtained from femora and tibiae of C57BL6 mice by flushing, and cells expressing one or more of the following surface proteins, CD16/CD32^+^, Gr1^+^, B220^+^, CD11b^+^, were depleted using immunomagnetic beads (sheep anti-rat IgG Dynabeads; Invitrogen, Paisley, UK). The remaining population was cultured in 2.6% serum-supplemented Stempro medium with 2 mM l-glutamine, penicillin/streptomycin, and 20 ng/mL murine stem cell factor (SCF) at 37°C under 5% CO_2_ for 2 days. Cells were then cultured for an additional 4 days in the presence of 20 ng/mL murine SCF and 100 ng/mL murine TPO [30,32]. FL cells were obtained from whole livers recovered from mouse fetuses between embryonic days 13 and 15. Single-cell suspensions were prepared by successive passage through 19-, 22-, and 25-gauge needles and cultured in Dulbecco's modified Eagle's medium supplemented with 10% fetal bovine serum, 2 mM l-glutamine, penicillin/streptomycin, and 100 ng/mL murine TPO for 5 days [31]. After 4 to 5 days of culture in the presence of TPO, the cell population was enriched in mature MKs using a 1.5%/3% bovine serum albumin (BSA) gradient under gravity (1*g*) for 45 minutes at room temperature. The proportion of mature MKs in the enriched population was estimated to be >60% (data not shown). To study the role of MAPKs in differentiation, inhibitors were added at the same time as TPO, and this was taken as the beginning of the differentiation process. The inhibitor was then given again after 24 hours at the same concentration (see Supplementary Fig. E1 [online only, available at www.exphem.org] for time course of differentiation and addition of reagents). An equivalent amount of dimethyl sulfoxide (<0.1%) or indomethacin (5 μM) were added to the control cells as stated in the text and figure legends.

#### MK biochemistry

After differentiation and BSA gradient isolation, mature MKs were harvested 4 hours at 37°C in serum-free media. Whole-cell lysates were prepared from MKs stimulated by TPO (50 ng/mL) or from BSA nonadherent and fibrinogen adherent MKs for the indicated times. Proteins were resolved on 4% to 12% NuPAGE Bis-Tris gradient gels and immunoblotted with primary antibodies and horseradish peroxidase−conjugated secondary antibody. Proteins were detected by enhanced chemiluminescence and autoradiography.

### Flow cytometry

Expression levels of CD34, GPIIb, GPIb, and CXCR4 were measured by flow cytometry using specific antibodies. Polyploidy of mature MKs isolated by BSA gradient was analyzed after anti-GPIIb labeling and DNA staining with propidium iodide. GPIIb-positive cells were gated to analyze DNA content. Samples were acquired using FACSCalibur flow cytometer and CellQuest software (Becton Dickinson, Oxford, UK) and analyzed using Summit v4.3 software (DAKO, Cambridge, UK).

### Cell migration assay

Chemotaxis was assessed using the Dunn chamber (Weber Scientific International, Teddington, UK) as described previously [32] and detailed in Supplementary Methods. To investigate the role of ERK1/2 and p38MAPK in migration, the Dunn chamber outer well was refilled with the medium containing stromal-cell−derived factor 1α (SDF1 α; 300 ng/mL) and the specific inhibitors, PD184161 (10 μM) or SB203580 (10 μM). Time-lapse images were digitally captured every minute for 4 hours using a Zeiss 20 × 1.40 NA plan-apochromat lens on a Zeiss Axiovert 200 inverted high-end microscope (Welwyn Garden City, UK) and a Hamamatsu Orca 285 cooled digital camera. Slidebook and Image J softwares were used to acquire and process images.

### Proplatelet formation assay

Fibrinogen-coated coverslips were prepared as described previously [33] by incubating overnight with fibrinogen (100 μg/mL) at 4°C and blocking with denatured BSA (5 mg/mL) in phosphate-buffered saline. Mature MKs were incubated for 15 minutes with MEK-ERK1/2 and p38MAPK pathway inhibitors, before being allowed to adhere to the fibrinogen-coated coverslip for 5 hours at 37°C. Adhered MKs were fixed and labeled with a fluorescein isothiocyanate (FITC)−anti-GPIIb antibody. Images were obtained using a Zeiss 40× oil immersion 1.40 NA plan-apochromat lens on a Zeiss Axiovert 200 M inverted high-end microscope and an Hamamatsu Orca 285 cooled CCD camera. The percentage of proplatelet-forming MKs and the area of proplatelet were analyzed. Slidebook and Image J softwares were used to acquire and process images.

### Quantitative real-time polymerase chain reaction (PCR) determination of transcript levels

Total RNA was isolated using the RNeasy Kit (Qiagen, Crawley, UK) according to the manufacturer's instructions and contaminating genomic DNA removed by treatment with RNAse-free DNAse kit (Qiagen). Single-stranded complementary DNA (cDNA) was prepared using SuperScript III reverse transcriptase with first-strand synthesis primed using oligo (dT). Quantitative real-time PCR was performed using SYBR Green PCR master mix. The reactions were carried in a Stratagene MX3000P system (Stratagene, Leicester, UK). Levels of CXCR4 amplified cDNA were normalized to the level of glyceraldehyde 3 phosphate dehydrogenase housekeeping control cDNA.

### Statistical analysis

Experiments were performed a minimum of three times and representative images are shown. Data is presented as mean ± standard error of mean and statistical analysis was conducted using two-tailed Student's *t-*test. A *p* value <0.05 was considered statistically significant.

## Results

### ERK1/2 is activated in response to TPO in primary BM- and FL-derived MKs

**Figure 1:**
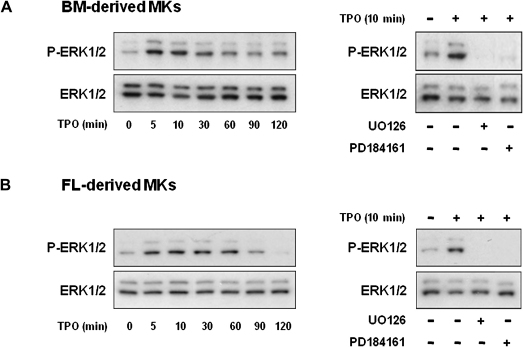
Time course of thrombopoietin (TPO)-induced extracellular regulated kinase (ERK)1/2 phosphorylation in primary murine megakaryocytes (MKs). Bone marrow (BM)− (**A**) and fetal liver (FL)−derived (**B**) mature MKs generated and isolated as described in Materials and Methods were harvested 4 hours at 37°C in serum-free media and stimulated with 50 ng/mL murine TPO for the indicated times. ERK1/2 phosphorylation was evaluated by immunoblot. To assess the effect of MEK inhibition on ERK1/2 activation**,** mature MKs were incubated with two specific MEK inhibitors, UO126 (10 μM) or PD184161 (10 μM) for 15 minutes followed by TPO stimulation for 10 minutes. Representative immunoblots of four independent experiments are shown.

Suspensions of mature BM- and FL-derived MKs were generated by culturing freshly isolated hematopoietic progenitor cells with TPO for 4 or 5 days, respectively, and subsequent purification using a BSA gradient. Different time points were used for the two populations, as the BM-derived MKs were found to consistently reach a higher level of maturity more rapidly that the FL-derived MKs. The ability of TPO to activate the MEK-ERK1/2 pathway in mature MKs was investigated by Western blotting. BM- and FL-derived MKs display a rapid and transient ERK1/2 phosphorylation upon stimulation by TPO, which peaks at 5 to 10 minutes, before returning to almost basal levels by 120 minutes (Figs. 1A and B; left-hand panels). The decline in ERK1/2 phosphorylation was noticeably more rapid in the BM-derived MKs. In both populations, phosphorylation of ERK2 (corresponding to the lower band) was more marked than ERK1. A similar set of observations were previously reported for murine BM-derived MKs by Rojnuckarin et al. [24]. These studies were also performed on immature MKs at day 2 of differentiation, by which time the maximal ploidy was 16N for the BM-derived cells and 8 N for the FL-derived cells. A similar profile of ERK1/2 activation to that in the mature cells was observed but with a more rapid decline to the baseline by 30 minutes in both cases (data not shown). Basal and TPO-induced phosphorylation of ERK1/2 was inhibited completely in the presence of the structurally distinct MEK inhibitors, U0126 and PD184161 (Figs. 1A and B; right-hand panels).

### ERK1/2-mediates differentiation of primary BM- and FL-derived MKs independent of p90 ribosomal S6 kinase (RSK)

**Figure 2:**
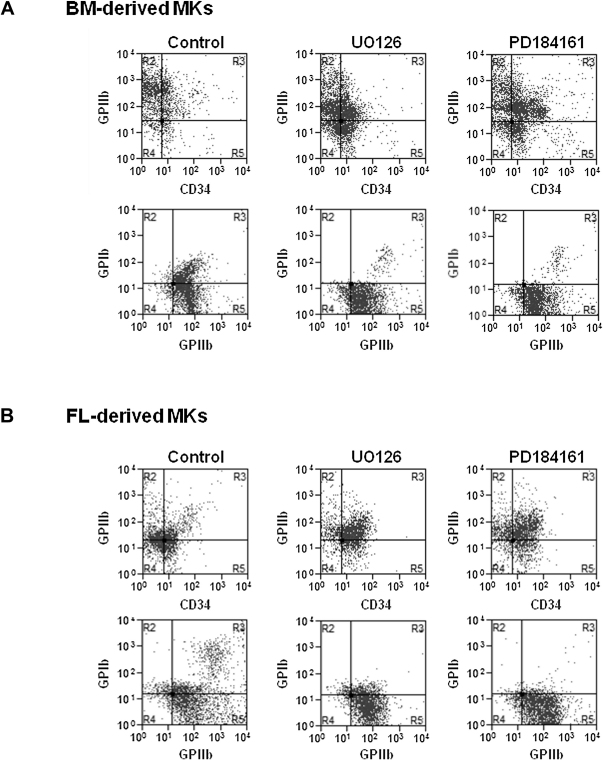
Effect of MEK inhibitors on cell surface expression markers. Progenitor cells isolated from bone marrow (BM) (**A**) and from fetal liver (FL) (**B**) were treated with the two MEK inhibitors U0126 (10 μM) or PD184161 (10 μM) along with thrombopoietin (TPO) (100 ng/mL) corresponding to the beginning of the differentiation process and the inhibitor dose repeated 24 hours later. Four or five days after addition of TPO, expression of CD34, GPIIb, and GPIb were evaluated by flow cytometry in the whole-cell population prior to bovine serum albumin (BSA) gradient (note that mature MKs express a high level of GPIIb and GPIb are designated as R3; R4 correspond to the isotype-matched controls). Representative profiles from four independent experiments are shown.

**Figure 3:**
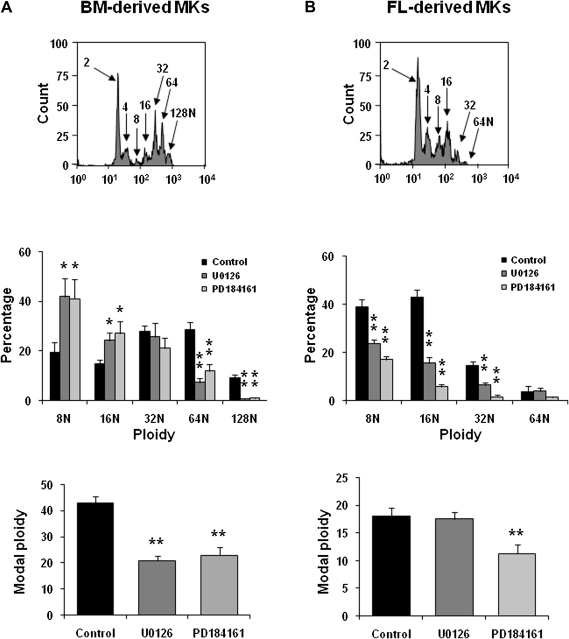
Effect of MEK inhibitors on bone marrow− (BM) and fetal liver (FL)−derived megakaryocyte (MK) differentiation. Progenitor cells isolated from BM (**A**) and FL (**B**) were treated with the two MEK inhibitors U0126 (10 μM) or PD184161 (10 μM) along with thrombopoietin (TPO) (100 ng/mL) corresponding to the beginning of the differentiation process and the inhibitor concentration was repeated 24 hours later. Four or five days after addition of TPO, propidium iodide fluorescence histogram on GPIIb expressing cells showing polyploidy distribution of MKs was evaluated by flow cytometry. Representative profiles from five independent experiments are shown. The percentage of cells with different polyploidy and the modal ploidy are mean ± standard error of mean of five independent experiments ^∗^*p* < 0.05; ^∗∗^*p* < 0.01, compared to control.

The effect of inhibition of the MEK-ERK1/2 pathway on BM- and FL-derived MK differentiation was investigated using the specific MEK inhibitors, U0126 and PD184161. Progenitors cells isolated from BM and FL were treated with the two inhibitors along with TPO at the beginning of the differentiation process. The inhibitor was also given 24 hours later as outlined in Supplementary Figure E1 (online only, available at www.exphem.org). Following differentiation of BM- and FL-derived cells for 4 or 5 days, respectively, the expression of the surface glycoproteins CD34, GPIIb, and GPIb, and the degree of polyploidy were examined. Flow cytometry profiles of the whole-cell population prior to separation on the BSA gradient revealed that the percentage of cells expressing GPIIb in the two models was unchanged after treatment with UO126 or PD184161 (Table 1 and Fig. 2), suggesting that ERK1/2 is not involved in the early stage of MK differentiation in either model. Conversely, inhibition of MEK-ERK1/2 induced, in both models, a significant increase in the percentage of cells expressing CD34 and a corresponding reduction in those expressing the late MK marker, GPIb (Table 1 and Fig. 2). Inhibition of the MEK-ERK1/2 pathway also reduced the overall DNA content (polyploidization) in the BSA gradient−enriched population of MKs, as analyzed by flow cytometry after anti-GPIIb labeling and propidium iodide staining (Figs. 3A and B). In the case of BM-derived MKs, this was associated with an increase in polyploidy levels between 8N and 16N and a reduction in cells with ploidy values of 64N or 128N (Fig. 3A). Although FL-derived MKs only generated ploidy levels up to 64N (Fig. 3B) compare to 128N for BM-derived MKs (Fig. 3A), inhibition of the MEK-ERK1/2 pathway caused a marked reduction in the percentage of cells with polyploidy levels between 8N and 64N (Fig. 3B). The modal ploidy of BM- and FL-derived MKs was also reduced following inhibition of the MEK-ERK1/2 pathway (Figs. 3A and B). These data are in agreement with the morphological analysis using May-Grunwald-Giemsa staining, which demonstrated a decrease in the MK size and in the number of nuclear lobes in treated cells compared to control cells (data not shown). Taken together, these results demonstrate that the MEK-ERK1/2 pathway plays a major role in the late stage differentiation of both BM- and FL-derived MKs and is critical for full polyploidization.

**Supplementary Figure E2:**
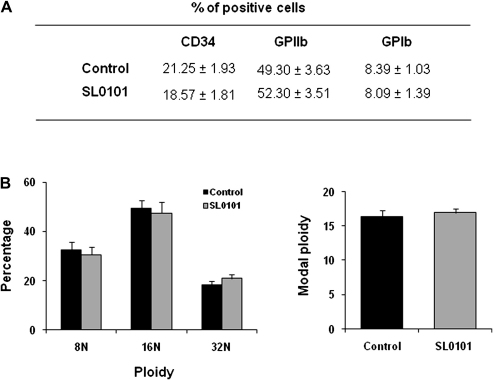
Effect of p90 ribosomal S6 kinase (RSK) inhibitor on fetal liver (FL)−derived megakaryocyte (MK) differentiation. Cells derived from FL were cultured with murine thrombopoietin (TPO) 100 ng/mL supplemented or not with SL0101 (10 μM) as described in Figures 1 and 3. (**A**) Expression of CD34, GPIIb, and GPIb were evaluated by flow cytometry of the whole-cell population prior to bovine serum albumin (BSA) gradient. The percentage of positive cells for the different markers is mean ± standard error of mean of three independent experiments. (**B**) FL-derived MKs after BSA gradient were stained with propidium iodide. Ploidy was evaluated by flow cytometry of MK culture grown in the presence of 100 ng/mL TPO in combination with methanol (control) or SL0101 (10 μM). GPIIb-positive cells were gated to analyze for DNA content. The percentage of cells with different polyploidy are mean from three independent experiments ± standard error of mean.

Many direct substrates of ERK1/2 are transcription factors and kinases, including the p90 ribosomal S6 kinases, RSK1−4. We therefore investigated the effect of a naturally occurring RSK inhibitor*,* SL0101 [34], on development of BM- and FL-derived MKs. In neither cell population did the inhibition of RSK using SL0101 (10 μM) induce a change in expression of the surface glycoproteins CD34, GPIIb, and GPIb, or in the degree of ploidy (Supplementary Fig. E2, online only, available at www.exphem.org), demonstrating that regulation of megakaryopoiesis by TPO is not mediated through activation of RSK.

### p38MAPK is not required for MK differentiation

**Supplementary Figure E3:**
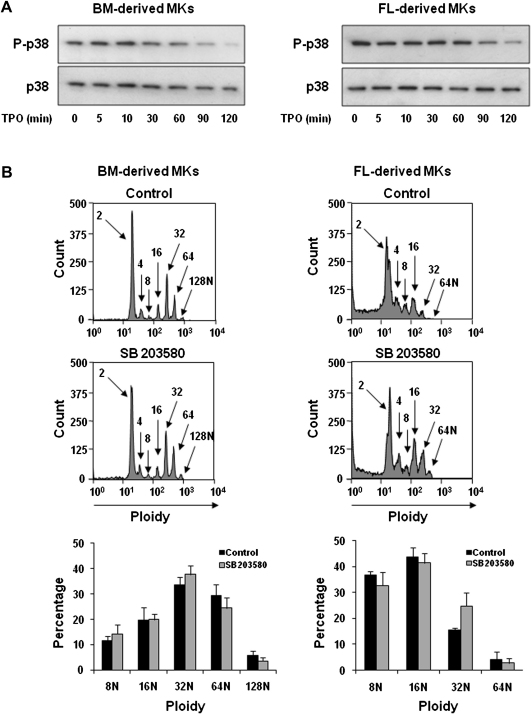

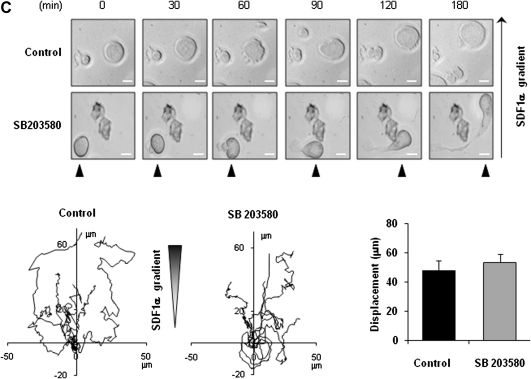
Time course of thrombopoietin (TPO)-induced p38 mitogen-activated protein kinase (MAPK) phosphorylation and effect of p38MAPK inhibitor on megakaryocyte (MK) endomitosis and migration. (**A**) Bone marrow (BM)- and fetal liver (FL)-derived mature MKs were harvested 4 hours at 37°C in serum-free media before stimulation with 50 ng/mL murine TPO for the indicated times. p38MAPK phosphorylation was evaluated by immunoblot. (**B**) Mature BM- and FL-derived MKs were cultured with murine 100 ng/mL TPO in the presence of indomethacin (5 μM) (control) or in combination with SB203580 (10 μM) as described in Figures 1 and 3. Ploidy was evaluated by flow cytometry (propidium iodide staining) of mature MKs. The percentage of cells with different polyploidy and the modal ploidy are mean ± standard error of mean of five independent experiments. Representative polyploidy profiles are shown (**C**) BM-derived mature MKs were allowed to migrate headed for the Dunn chamber outer well containing stromal-cell-derived factor 1 α (SDF1α) and SB203580 (10 μM) for 4 hours. The net translocation distance (displacement from the start to the endpoint) of each cell is represented and is a total of four independent experiments.

A similar set of studies to those described here were carried out in BM- and FL-derived MKs to investigate a possible role for the p38MAPK pathway in differentiation. p38MAPK is constitutively phosphorylated in both BM- and FL-derived MKs and undergoes a decrease in phosphorylation upon TPO stimulation at 30 and 90 minutes, respectively (Supplementary Fig. E3A, online only, available at www.exphem.org). In view of the constitutive activation of p38MAPK, we investigated the effect of the p38MAPK inhibitor, SB203580, on MK differentiation. These experiments were performed alongside controls in the presence of indomethacin in view of the ability of SB203580 to block cyclooxygenase [35]. Treatment with SB203580 (10 μM) using the same protocol as that for inhibition of the MEK-ERK1/2 pathway had no effect on expression of CD34, GPIb, and GPIIb markers, or on the degree of ploidy in either BM or FL-derived MKs relative to controls (data not shown and Supplementary Fig. E3B, online only, available at www.exphem.org). These results demonstrate that p38MAPK is not involved in primary murine MK differentiation.

### ERK1/2 is involved in MKmigration in response to SDF1α gradient

It is recognized that as MKs mature and differentiate in the BM, they migrate to sinusoidal BM endothelial cells, where they form transendothelial projections called proplatelets [1,2]. In turn, these give rise to platelets, either before or after release of proplatelets into the circulation. SDF1α is the major chemotactic agent involved in regulation of MK migration to the BM niche [36]. This stage of megakaryopoiesis can be followed in vitro by monitoring migration of MK on fibronectin, a major extracellular matrix component in BM [37], toward a gradient of SDF1α using a Dunn chamber. We have used this approach to compare the ability of mature MKs grown from BM and FL progenitors for 4 days or 5 days, respectively, to migrate along a SDF1α gradient.

**Figure 4:**
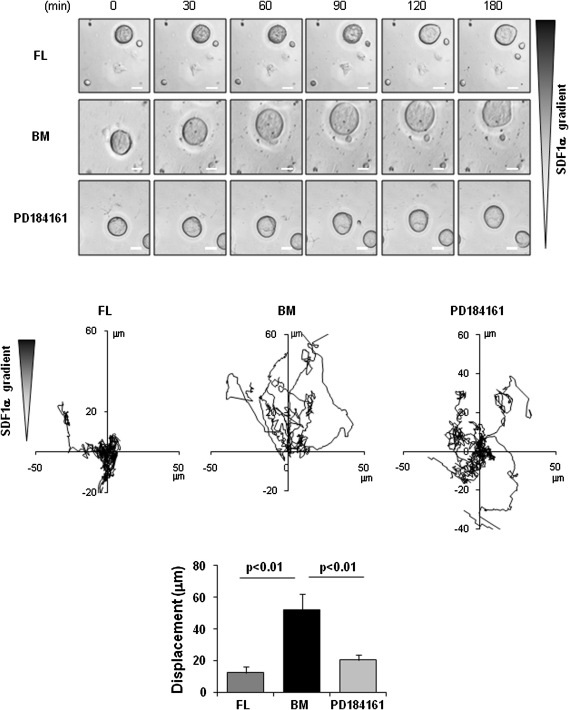
Megakaryocyte (MK) migration toward a stromal-derived factor −1α (SDF1α) gradient. Bone marrow (BM)− and fetal liver (FL)−derived mature MKs adherent on fibronectin (20 μg/mL)-coated coverslip were allowed to migrate toward an SDF1α gradient over 4 hours within the Dunn chamber as described in Materials and Methods. PD184161 (10 μM) was added to the Dunn chamber outer well containing SDF1α (300 ng/mL). Representative differential interference contrast images from four independent experiments of primary MKs exposed to SDF1α gradient are shown (scale bar: 10 μm). The net translocation distance (displacement from the start to the endpoint) of each cell is represented.

**Figure 5:**
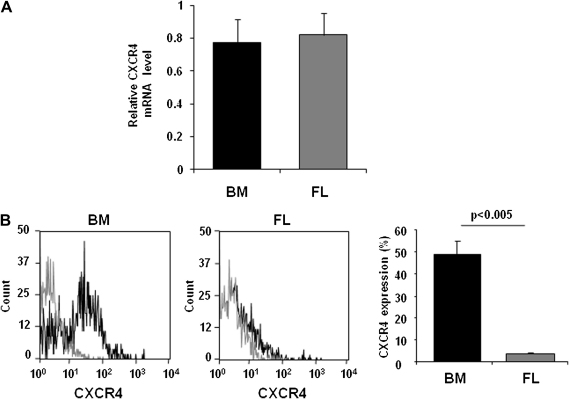
CXCR4 expression in fetal liver (FL)− and bone marrow (BM)−derived megakaryocytes (MKs). (**A**) Relative CXCR4 mRNA expression of BM- and FL-derived MKs normalized to the glyceraldehyde 3 phosphate dehydrogenase housekeeping complementary DNA was evaluated by real-time quantitative polymerase chain reaction. Data are presented as mean ± standard error of mean of three independent experiments. (**B**) CXCR4 surface expression of BM- and FL-derived mature MKs was analyzed by flow cytometry using a fluorescein isothiocyanate (FITC)−conjugated anti-CXCR4 antibody. Gray line indicates relevant antibody control; black line, FITC-conjugated anti-CXCR4 antibody. Representative profiles from nine independent experiments are shown. The percentage of cells expressing CXCR4 were quantified and presented as mean ± standard error of mean.

In agreement with our previous results [32], BM-derived MKs migrate toward a gradient of SDF1α, whereas, in contrast, FL-derived MKs do not migrate under the same conditions (Fig. 4 and Supplementary Fig. E4, videos 1 and 2, online only, available at www.exphem.org). To investigate a possible explanation for this difference, we measured the transcript and the protein cell surface levels of the SDF1α receptor, CXCR4, in both MK populations. CXCR4 transcript level analyzed by real-time quantitative PCR were comparable in both models (Fig. 5A), whereas flow cytometry showed a robust surface expression of CXCR4 in BM-derived MKs, but a negligible level on the FL-derived mature MK surface (Fig. 5B), thereby accounting for the inability of these cells to migrate to SDF1α. A negligible level of CXCR4 was also found on immature BM- and FL-derived cells (data not shown). BM-derived progenitor cells, therefore, express an increase in CXCR4 surface as megakaryocytic differentiation proceeds, which are not observed in the FL cell model. Consequently, we restricted our investigation of the role of ERK1/2 and p38MAPK in migration to the population of BM-derived MKs. Administration of the MEK inhibitor PD184161 (10 μM) to the Dunn chamber caused the cells to move relatively short distances and to more readily change direction compared to control cells (Fig. 4 and Supplementary Fig. E4, video 3, online only, available at www.exphem.org), suggesting a lack of directed chemotaxis in response to SDF1α. In contrast, inhibition of p38MAPK using SB203580 had no effect on MK migration (Supplementary Fig. E3C and Fig. E4, videos 4 and 5, online only, available at www.exphem.org). These results demonstrate a critical role for ERK1/2 activation in migration of BM-derived MKs.

### ERK1/2 is required for proplatelet formation

**Figure 6:**
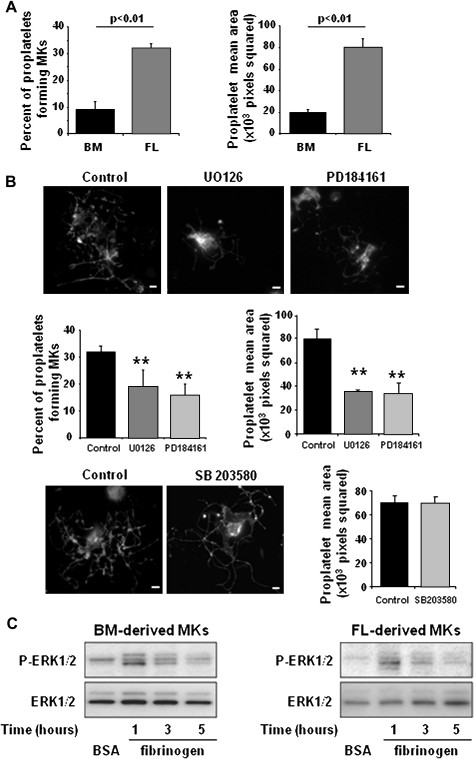
Effect of extracellular signal-regulated kinase (ERK)1/2 and p38 mitogen-activated protein kinase (MAPK) inhibition on proplatelet formation. (**A**) Bone marrow (BM)− and fetal liver (FL)−derived mature megakaryocytes (MKs) were allowed to adhere on fibrinogen (100 μg/mL) coated coverslips for 5 hours at 37°C, fixed and labeled with a fluorescein isothiocyanate (FITC)−anti-GPIIb antibody. Percent of proplatelet-forming MKs was analyzed and proplatelet areas were quantified using Image J software. Data are presented as mean ± standard error of mean (SEM) of three independent experiments. (**B**) FL-derived mature MKs preincubated with U0126 (30 μM), PD184161 (10 μM), or SB203580 (10 μM) were allowed to adhere on fibrinogen, fixed and labeled with a FITC−anti-GPIIb antibody. Percent of proplatelet-forming MKs and proplatelet areas were quantified and data are presented as mean ± SEM of three independent experiments. Representative GPIIb immunofluorescent images of proplatelet-forming MKs are shown (scale bar: 10 μm). (**C**) ERK1/2 phosphorylation was evaluated by immunoblot from whole cell lysates prepared from bovine serum albumin (BSA) nonadherent and fibrinogen adherent MKs for the indicated times. Representative immunoblots of three independent experiments are shown.

MKs were grown to maturity from BM- and FL-derived progenitors for 4 days and 5 days respectively, purified by BSA gradient and then allowed to generate proplatelets on an immobilized fibrinogen surface over a period of 5 hours. Although both populations of MKs were able to generate proplatelets, approximately three times the number of proplatelet-forming MKs were observed in the FL-derived population, correlated to an increase of the total surface area of the proplatelet network (Fig. 6A). As a consequence, we restricted our investigation of the role of the MEK-ERK1/2 pathway in proplatelet formation to the FL-derived MKs. Inhibition of this pathway using two specific inhibitors, U0126 and PD184161, reduced the number of proplatelet-forming MKs by >50%, with a corresponding reduction in proplatelet area (Fig. 6B). In contrast, inhibition of p38MAPK using SB203580 had no significant effect on the formation of proplatelets in comparison to the indomethacin-treated control (Fig. 6B). Together, these results suggest that ERK1/2 activation is required in the process of proplatelet formation, whereas p38MAPK is not involved. Interestingly, we observed ERK1/2 phosphorylation in both MK populations at 1 hour after their adhesion to immobilized fibrinogen, which returns to basal level by 5 hours (Fig. 6C).

## Discussion

In the present study, we have compared the ability of BM- and FL-derived MKs to differentiate, migrate toward a gradient of SDF1α, and generate proplatelets. The role of MEK-ERK1/2 and p38MAPK pathways in these processes has been investigated. The results demonstrate a critical role for the ERK1/2 pathway, but not p38MAPK, in all of these processes, as well as several differences in the behavior of the two cell populations, of which the most notable was the failure of the FL-derived cells to migrate toward a gradient SDF1α due to a negligible level of CXCR4 receptor surface expression.

MK differentiation is characterized by expression of specific megakaryocytic surface glycoproteins and nuclear maturation, resulting in large polyploidy cells. In this study, the role of MEK-ERK1/2 pathway in the differentiation of BM- and FL-derived MK was investigated using relatively low concentrations of these two structurally distinct MEK inhibitors, UO126 and PD186141. Inhibition of MEK-ERK1/2 pathway is sufficient to induce an increase of expression of the immature marker CD34 and a decrease in the late MK commitment marker, GPIb, without affecting expression of the early megakaryocytic marker GPIIb. Furthermore, inhibition of the MEK-ERK1/2 pathway causes a leftward shift of the ploidy of the cells, showing a critical requirement of MEK-ERK1/2 pathway in late stage of MK differentiation. In contrast, the p38MAPK does not contribute to MK differentiation. Indeed, Miyazaki et al. has shown that, in cord blood primary MK, p38MAPK is required for TPO-induced erythroid differentiation and not for MK differentiation [23].

During megakaryopoiesis, differentiating MKs migrate within the complex BM stromal environment from the endosteal niche to the BM sinusoidal endothelial cells, the site of platelet formation. The chemokine SDF1α augments this motility and promotes the interaction of MKs with the BM vascular niche and, therefore, supports thrombopoiesis [36]. Interestingly, we show that, contrary to BM-derived MKs, MKs derived from FL are not able to migrate in response to SDF1α because of a negligible level of CXCR4 cell surface expression, despite comparable level expression of CXCR4 gene in both models. This suggests a different regulation of megakaryopoiesis in these two models. One potential physiological explanation for this is that MKs in the liver do not need to migrate to release platelets because of a highly vascular environment. In contrast, within the complex BM microenvironment, MKs needs to migrate from the proliferative osteoblastic niche to the capillary-rich vascular niche to release platelets. Previous studies have revealed that MAPKs are important factors in the regulation of cell migration in numerous cell types in response to cell matrix proteins, growth factors, or cytokines [38]. In this study, we demonstrate a critical role for ERK1/2, but not p38MAPK, in MK movement and chemotaxis in response to SDF1α. The defective migration might be explained by the altered polarization of the SDF1α receptor CXCR4, as shown previously [32]. Moreover, ERK1/2 may govern cell movement by phosphorylating the myosin light-chain kinase, the protease calpain, or the focal adhesion kinase. These phosphorylations regulate the dynamics of focal adhesions and the reorganization of cytoskeleton, which are critical for cell migration [39−41]. Further studies will focus upon elucidating the precise mechanisms by which ERK1/2 control cell migration.

The role of MEK-ERK1/2 and p38MAPK pathways in the terminal stage of megakaryopoiesis, proplatelet formation, was also investigated. An increased proportion of FL-derived MKs generate proplatelets compared to BM-derived MKs, perhaps reflecting the need to generate a large number of platelets in parallel to development of the vasculature in the fetus, whereas in contrast, the role of BM-derived MKs is to maintain a steady-state platelet production. Interestingly, in culture conditions, BM-derived MKs present a higher ploidy, but form fewer proplatelets than FL-derived MKs, suggesting that a higher threshold of MK maturation may be necessary to achieve proplatelet formation in BM. The present study also demonstrates that MEK-ERK1/2 pathway, but not p38MAPK, is required for proplatelet generation. It is now well-established that dynamic regulation of microtubules is essential for proplatelet formation [42−44] and that microtubule-associated protein, which are well-characterized substrates of MAPKs [45,46], play a major role in microtubule dynamics [47]. One of the potential mechanisms implicating ERK1/2 in proplatelet formation is therefore the regulation of the phosphorylation states of the microtubule-associated proteins and subsequent organization of the microtubule network.

In summary, we provide evidence for the critical role of the MEK-ERK1/2 pathway, but not p38MAPK, in MK differentiation, migration, and proplatelet formation, and describe several differences in the behavior of the two primary MK populations from BM and FL. Further experiments are required to identify and confirm the roles of the substrates of ERK1/2 that underlie these effects.

## Acknowledgments

This work was supported by the following British Heart Foundation (London, United Kingdom) grants: PG/07/041/22896 and PG/06/129/21681. S.P.W. holds a BHF Chair (CH/03/003). We thank Drs. Cedric Ghevaert, Tarvinder Dhanjal and Yotis Senis for critical review of this manuscript. We wish to thank Dr. Steve Thomas for help in establishing the methodology for the MK cultures and proplatelet assay and for a critical review of this manuscript. We thank Dr. Emilie Dasse for providing help for the quantitative real-time PCR methodology.

## Conflict of Interest

The authors state that they have no conflict of interest.

## Figures and Tables

**Table 1:** Effect of MEK inhibitors on cell surface expression markers

	% of positive cells		
	CD34	GPIIb	GPIb
BM cells			
Control	24.3 ± 5.2	76.9 ± 6.3	13.4 ± 0.6
UO126	54.6 ± 3.2 ∗∗	76.2 ± 2.1	0.4 ± 0.3 ∗∗
PD184161	57.0 ± 6.0 ∗∗	81.4 ± 3.7	1.6 ±0.7 ∗∗
FL cells			
Control	15.4 ± 2.8	55.8 ± 2.7	7.5 ± 5.2
UO126	30.6 ± 0.9 ∗∗	52.0 ± 4.9	1.6 ± 1.3 ∗∗
PD184161	36.4 ± 4.8 ∗∗	66.3 ± 10.0	0.9 ± 0.8 ∗∗

Percentage of positive cells was determined by flow cytometry using specific CD34, GPIIb, and GPIb antibodies on the whole population prior to bovine serum albumin (BSA) gradient. Mean ± standard error of mean of four independent experiments are shown. BM = bone marrow; FL = fetal liver.

∗∗ *p* < 0.01, when compared with control.

## Supplementary methods

### Reagents, antibodies, and suppliers

Recombinant murine TPO, murine stem cell factor, and murine SDF1a were purchased from PeproTech (London, UK). Sheep anti-rat IgG Dynabeads, biotin-conjugated rat anti-mouse CD45R/B220, purified rat anti-mouse CD16/CD32, FITC- or PE-conjugated anti-mouse GPIIb and PE-conjugated anti-mouse GPIb antibodies were from BD Pharmingen (Oxford, UK). Anti-mouse Ly-6G, biotin anti-mouse CD11b and FITC-conjugated anti-mouse CD34 antibodies were from eBioscience (Wembley, UK). Anti-mouse CD49e antibody was from Serotec (Oxford, UK). Stempro medium and Dulbecco’s modified Eagle’s medium were from GIBCO, Invitrogen (Paisley, UK). The p38MAPK inhibitor, SB203580, was obtained from Calbiochem (Nottingham, UK). The MEK inhibitor PD184161 was a gift from Pfizer Global Research and Development (Sandwich, UK). The MEK inhibitor U0126 and the polyclonal antibody directed against the phosphorylated form of p38MAPK were purchased from Promega Corp. (Madison, WI, USA). The monoclonal anti-phospho-ERK1/2, the polyclonal anti-p38MAPK, the goat anti-rabbit-horseradish peroxidase and goat anti-mouse-horseradish peroxidase P antibodies were obtained from Santa Cruz Biotechnology (Santa Cruz, CA, USA). SL0101, a specific RSK inhibitor, was purchased from Toronto Research Chemicals (North York, Canada). FITC conjugated anti-mouse CXCR4 antibody was from R&D Systems (Abingdon, UK). All other reagents were purchased from Sigma-Aldrich (St Louis, MO, USA).

### Flow cytometry

Expression levels of GPIIb, GPIb, and CD34 were measured by flow cytometry. Aliquots of TPO-culture whole cell population before BSA gradient were stained with 2.5 mL FITC-anti-GPIIb, PE-anti-GPIIb, FITC-anti-GPIb, or FITC-anti CD34 antibodies in 50 mL 1% BSA/PBS for 30 minutes at room temperature. Relevant isotype-matched controls directly conjugated to FITC or PE were used as controls. Samples were acquired using FACSCalibur flow cytometer and CellQuest software (Becton Dickinson, Oxford, UK) and analyzed using Summit v4.3 software (DAKO, Cambridge, UK).

### MK ploidy

Following labelling with FITC-anti-GPIIb, mature MKs were fixed with 1% paraformaldehyde for 45 minutes at 4°C, and then washed with PBS. MK polyploidy was analyzed following DNA staining with 0.01 mg/mL PI in solution containing 2 mM MgCl_2_, 0.05% saponin, and 10 U/mL RNAse A. Samples were acquired using FACSCalibur flow cytometer and CellQuest software (Becton Dickinson, Oxford, UK) and analyzed using Summit v4.3 software (DAKO, Cambridge, UK). DNA plots were taken from GPIIb expressing cells only.

### Cell migration assay

Chemotaxis was assessed using the Dunn chamber (Weber Scientific International, Teddington, UK). BM-derived mature MK suspension was applied on fibronectin-coated (20 mg/mL) coverslips in complete medium and left at 37°C for 1 hour for cells to adhere. The coverslip was then placed directly onto the Dunn chamber where both the inner and the outer wells were filled with medium. Using sterile filter paper, the outer well was drained to leave the inner well and the region over the bridge filled with medium. The outer well was then refilled with the same medium containing 300 ng/mL SDF1a. The Dunn chamber was placed in a humidified chamber on a heated stage at 37°C within an inverted microscope. Time-lapse images were digitally captured every minute for 4 hours using a Zeiss 40 × oil immersion 1.40 NA plan-apochromat lens on a Zeiss Axiovert 200M inverted high-end microscope (Welwyn Garden City, UK) and an Hamamatsu Orca 285 cooled CCD camera.Zeiss Axiovert 200 inverted high-end microscope and Hamamatsu Orca 285 cooled digital camera. To track migration paths, a series of images were analyzed using Slidebook and Image J software, and the data were plotted with Microsoft Excel. Net translocation distance was determined as the straight distance between the endpoints during the 4 hour-period.

Supplementary Figure E4. Video 1 Fetal liver (FL)-derived megakaryocytes (MKs) do not migrate toward a stromal-derived factor − 1α (SDF1α) gradient over a fibronectin-coated surface. FL-derived MKs plated on a fibronectin-coated surface were imaged in real-time migrating towards an SDF1α gradient. Images were captured by differential interference contrast microscopy (Zeiss Axiovert 200 microscope; Hamamatsu Orca 285 cooled digital camera). The time in minutes is indicated in the top left corner. Representative video from four independent experiments are shown.

Supplementary Figure E4. Video 2 Bone marrow (BM)−derived megakaryocytes (MKs) migrate toward a stromal-derived factor − 1α (SDF1α) gradient over a fibronectin-coated surface. BM-derived MKs plated on a fibronectin-coated surface were imaged in real-time migrating toward an SDF1α gradient. The time in minutes is indicated in the top left corner. Representative video from four independent experiments are shown.

Supplementary Figure E4. Video 3 Effect of the MEK−extracellular signal-regulated kinase (ERK)1/2 pathway inhibition on bone marrow (BM)-derived megakaryocyte (MK) migration toward a stromal-derived factor − 1α (SDF1α) gradient. BM-derived MKs plated on a fibronectin-coated surface were imaged in real-time migrating toward an SDF1α gradient in the presence of PD184161 (10 μM). The time in minutes is indicated in the top left corner. Representative video from four independent experiments are shown.

Supplementary Figure E4. Video 4 Effect of indomethacin on bone marrow (BM)−derived megakaryocyte (MK) migration toward a stromal-derived factor − 1α (SDF1α) gradient. BM-derived MKs plated on a fibronectin-coated surface were imaged in real-time migrating toward an SDF1α gradient in the presence of indomethacin (5 μM). The time in minutes is indicated in the top left corner. Representative video from four independent experiments are shown.

Supplementary Figure E4. Video 5 Effect of p38 mitogen-activated protein kinase (MAPK) inhibitor bone marrow (BM)−derived megakaryocyte (MK) migration toward a stromal-derived factor − 1α (SDF1α) gradient. BM-derived MKs plated on a fibronectin-coated surface were imaged in real-time migrating toward a SDF1α gradient in the presence of indomethacin (5 μM) and SB203580 (10 μM). The time in minutes is indicated in the top left corner. Representative video from four independent experiments are shown.
