# Supplementary figures and images for: Critical role for ERK1/2 in bone marrow and fetal liver–derived primary megakaryocyte differentiation, motility, and proplatelet formation
Source: Exp Hematol. 2009 Oct;37(10):1238–1249.e5. doi: 10.1016/j.exphem.2009.07.006 (PMC2755112; doi:10.1016/j.exphem.2009.07.006)

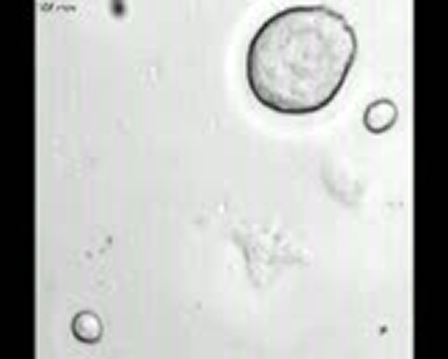

Supplement: Supplementary Figure E4. Video 1 — Fetal liver (FL)-derived megakaryocytes (MKs) do not migrate toward a stromal-derived factor − 1α (SDF1α) gradient over a fibronectin-coated surface. FL-derived MKs plated on a fibronectin-coated surface were imaged in real-time migrating towards an SDF1α gradient. Images were captured by differential interference contrast microscopy (Zeiss Axiovert 200 microscope; Hamamatsu Orca 285 cooled digital camera). The time in minutes is indicated in the top left corner. Representative video from four independent experiments are shown. [file mmc1.jpg]

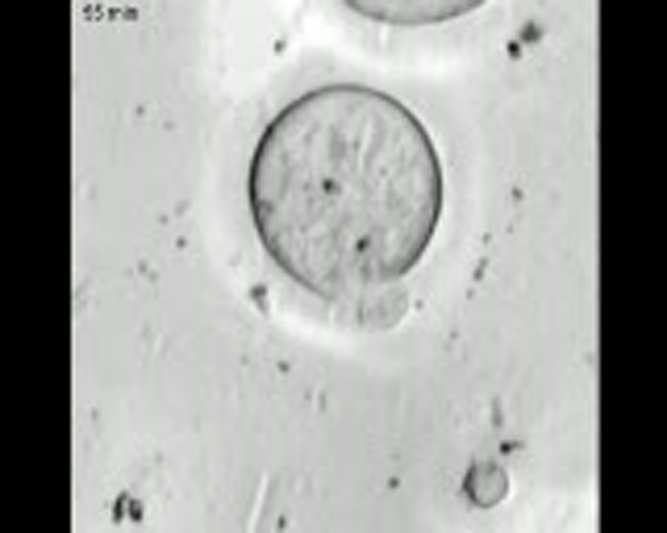

Supplement: Supplementary Figure E4. Video 2 — Bone marrow (BM)−derived megakaryocytes (MKs) migrate toward a stromal-derived factor − 1α (SDF1α) gradient over a fibronectin-coated surface. BM-derived MKs plated on a fibronectin-coated surface were imaged in real-time migrating toward an SDF1α gradient. The time in minutes is indicated in the top left corner. Representative video from four independent experiments are shown. [file mmc2.jpg]

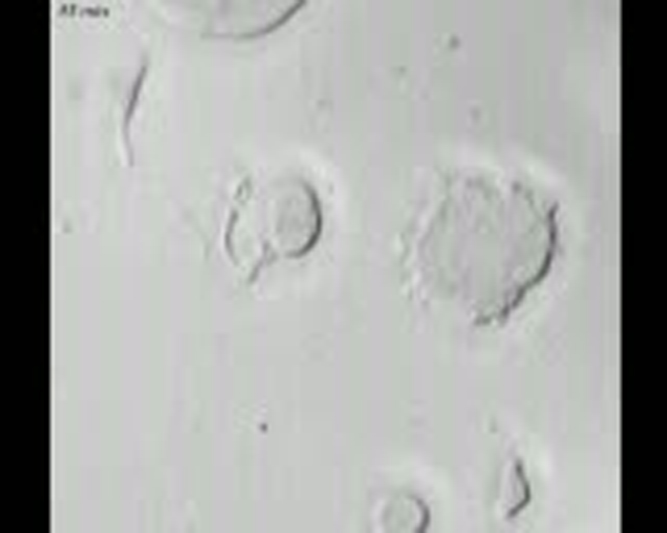

Supplement: Supplementary Figure E4. Video 4 — Effect of indomethacin on bone marrow (BM)−derived megakaryocyte (MK) migration toward a stromal-derived factor − 1α (SDF1α) gradient. BM-derived MKs plated on a fibronectin-coated surface were imaged in real-time migrating toward an SDF1α gradient in the presence of indomethacin (5 μM). The time in minutes is indicated in the top left corner. Representative video from four independent experiments are shown. [file mmc4.jpg]

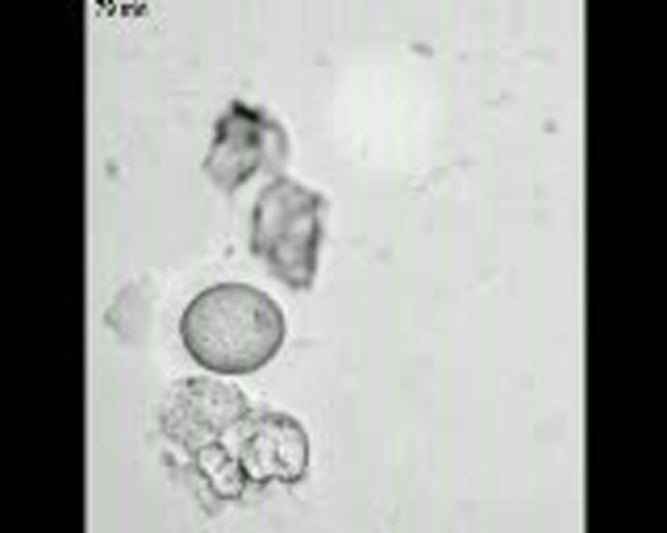

Supplement: Supplementary Figure E4. Video 5 — Effect of p38 mitogen-activated protein kinase (MAPK) inhibitor bone marrow (BM)−derived megakaryocyte (MK) migration toward a stromal-derived factor − 1α (SDF1α) gradient. BM-derived MKs plated on a fibronectin-coated surface were imaged in real-time migrating toward a SDF1α gradient in the presence of indomethacin (5 μM) and SB203580 (10 μM). The time in minutes is indicated in the top left corner. Representative video from four independent experiments are shown. [file mmc5.jpg]
